# Basic research on layered geopolymer composites with insulating materials of natural origin

**DOI:** 10.1038/s41598-024-63442-9

**Published:** 2024-05-31

**Authors:** Agnieszka Bąk, Janusz Mikuła, Inna Oliinyk, Michał Łach

**Affiliations:** 1 Faculty of Material Engineering and Physics, Chair of Material Engineering and Physics, Cracow University of Technology, Jana Pawła II 37, 31-864 Cracow, Poland; 2 Department of Materials Science and Engineering, Pryazovsky State Technical University, Mariupol, Ukraine

**Keywords:** Geopolymer, Natural fiber, Alkaline activation, Insulation material, Construction, CO_2_ reduction, Climate-change ecology, Environmental economics, Characterization and analytical techniques, Design, synthesis and processing, Microscopy, Civil engineering

## Abstract

New restrictions on carbon dioxide emissions and electricity consumption are currently being introduced around the world. Innovative solutions are being adopted in many countries to reduce CO_2_ emissions and material and energy consumption. The present work is related to the study of innovative binders based on geopolymers for the production of layered building envelopes. The binders are reinforced with composite bars and containing fibers of natural origin. The natural materials used to produce the samples are completely biodegradable. A 10-mol sodium hydroxide solution with an aqueous solution of sodium silicate was used for alkaline activation of geopolymers. The purpose of the study was to compare and determine the insulating properties of natural fiber-based materials such as coconut mat, jute felt, hemp felt, flax felt, flax wool, hemp wool, flax-jute wool, and to determine the effect of these materials on geopolymer composites, in which 4 layers of natural insulating materials were used, and the composites were reinforced by fiberglass bars. The publication presents the results of physicochemical studies of geopolymerization precursors and natural insulating materials, studies of thermal properties of fibers, mats, felts and wools, morphology of fiber structure and texture, as well as physical and thermal properties of finished multi-layer partitions. The results indicate the great potential of these materials in prefabrication and structural-insulation applications. The fabricated composites using 4 layers of natural fibers showed improved thermal conductivity by as much as 40% (reduced thermal conductivity from 1.36 W/m × K to about 0.8 W/m × K). The work may have future applications in energy-saving and low-carbon construction.

## Introduction

**Figure 1:**
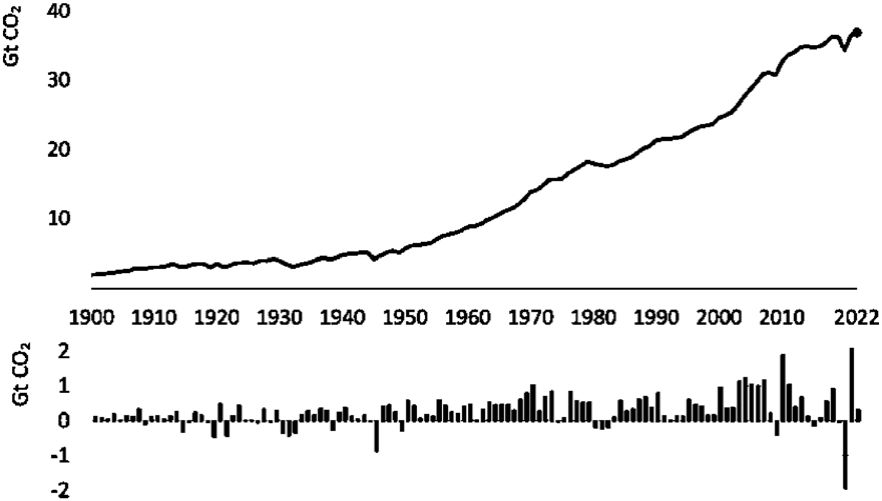
The growth of carbon dioxide emissions between 1900 and 2022.

Scientific research over the years has confirmed that the main cause and driving force behind climate change is the greenhouse effect, a phenomenon manifested by an increase in the temperature of the planet’s surface through greenhouse gases present in its atmosphere. The group of greenhouse gases includes methane, nitrous oxide, fluorinated greenhouse gases and carbon dioxide (CO_2_). The last of these comes from human activities and contributes the most to global warming^1–4^. The current concentration of carbon dioxide is the highest it has been in 14 million years and is steadily increasing. Figure 1 shows a graph showing the increase in global CO_2_ emissions from energy and industrial processes between 1900 and 2022^5–7^. This picture illustrates that the increase in CO_2_ emissions is very evident and, in view of this, new solutions are needed to reduce the effects of climate change. New ideas should contribute to climate protection and positively influence the possibility of adaptation and mitigation to climate change.

The building sector (construction industry) is one of the key areas where a number of measures can be introduced to reduce CO_2_ emissions. The operation of buildings accounts for 30% of global final energy consumption and 26% of global energy-related emissions (8% are direct emissions from buildings and 18% indirect emissions from the production of electricity and heat used in buildings). According to IEA (International Energy Agency) data, direct emissions from the buildings sector fell in 2022 compared to the previous year, despite extreme temperatures causing an increase in heating-related emissions in some regions. Energy consumption in the buildings sector increased by about 1% in 2022. Increasingly stringent minimum performance standards and building energy standards are being developed in many countries. The use of increasingly efficient and renewable building technologies is accelerating, but the sector needs faster change to achieve a net-zero emissions scenario by 2050 (NZE). The current decade is critical for implementing the measures required to achieve the goals of having all new buildings and 20% of existing buildings ready for zero-carbon technology by 2030^8^.

Examples of regulations aimed at reducing CO_2_ emissions include the REPowerEU plan and Fit for 55^9,10^. In the construction industry, the concept of sustainable construction is important^11^, where special emphasis is placed on the environmental impact of materials and products. It is necessary not only to reduce carbon emissions, but also to use natural resources sparingly^12–14^. CO_2_ emissions are associated not only with inadequate and inefficient insulation of buildings, but also with the production of building materials. Available reports on the state of the environment, as well as scientific studies, show that the entire construction industry generates as much as 38% of global CO_2_ emissions. According to various estimates presented in climate policy reports, the cement industry is responsible for generating about 5–6% of global CO_2_ emissions. It should be remembered, however, that when analyzing the construction industry from this angle, it is not only the cement industry that generates significant amounts of CO_2_, but also the construction chemicals and insulation materials used in the construction of buildings are responsible for these emissions^15,16^. As reported in the IEA report, the direct CO_2_ emissions intensity from cement production has remained stable over the past five years, but has increased slightly (by 1%) in 2022. In contrast, it is necessary to achieve an annual decline in CO_2_ intensity of 4% by 2030 for the sector in order to achieve a net zero emissions scenario by 2050 (NZE). For example, reducing the ratio of clinker to cement through the use of clinker substitutes, continuously improving energy efficiency, adopting low-carbon fuels, improving material efficiency, and implementing innovative technologies such as carbon capture and storage (CCS) are cited as ways to achieve this goal^17^.

A practical solution to improve cement substitutes, produce low-carbon binders, and enhance energy efficiency involves using materials that emit significantly lower CO_2_ and utilize hard-to-manage energy industry waste. In addition, these materials can provide effective insulation through the use of renewable biobased materials. We are talking about geopolymer materials in the form of composites with natural fibers or other renewable materials often of waste origin. Many studies have been written on the use of natural materials in building composites. The use of biomass waste and plant fibers as fillers for traditional binders has shown environmental promise for reducing overall energy consumption and carbon emissions in the construction sector. Coffee waste, for example, can be used, as studies have shown that adding as little as 5% wt. SCG (spent coffee grounds) can improve the thermal insulation of mortars by 58% and reduce their thermal diffusivity by 34%. Therefore, SCGs represent a promising bio-waste aggregate for developing low-impact thermal insulation mortars on an industrial scale^18^. Also, tea waste can be an attractive material, as it has been proven that the addition of ST (spent tea) leads to a significant decrease in thermal conductivity (67%), thermal diffusivity (57%), density (24%). Addition of up to 2.5% wt. ST meets the structural requirements of lightweight concretes (> 3.5 MPa) and can be used in wall structures, while a composite containing 7.5% wt. ST can be used for insulation purposes^19^. Another example of an interesting material could be waste from olive oil production. The use of olive pomace has been proven to reduce thermal conductivity by 55.68% and increase compressive strength by 81.7% of composites^20^.

Studies in Morocco have shown that the use of 4% by weight of straw in plasters will reduce their thermal conductivity by 68%. Energy simulations conducted in six climate zones in Morocco using the “Type56a” module of the TRNSYS software concluded that implementing the proposed solution could lead to a 13% reduction in greenhouse gas emissions from buildings, and annual costs could be reduced by about 13% in all climate zones in Morocco^21^. Studies were also conducted for the addition of Pennisetum Setaceum (PS) fibers and their effect on the thermomechanical properties of adobe clay was determined. Experiments have shown that adobe clay with the addition of PS fibers can be used as a green thermal mass component in modern buildings, contributing to thermal insulation^22^. The topic of using natural fibers in innovative geopolymer materials is well known and widely reported in the literature^23–26^.

Although the use of biomass-based wastes or natural fibers has a positive impact on the properties of construction materials, the issue is not without drawbacks or limitations. Natural fibers in a cement or geopolymer matrix can degrade significantly^27–29^. As outlined in numerous scientific papers^30–35^, the addition of fibers to binders may require appropriate treatment to increase their durability or improve the quality of the bond between the fibers and the matrix. Cellulose is the compound responsible for the fiber’s strength, while lignin and hemicellulose are responsible for its low durability, so it is often necessary to carry out treatments, most often with alkaline solutions, to improve the adhesion of the matrix to the fiber in the contact area. It has been confirmed, for example, that fibers such as açai, jute, sisal, bamboo and curaua can find use after appropriate treatment as a filler in cement matrices^36^.

The article summarizes the mechanism of alkaline degradation, factors affecting the degradation process, and the pathway for improving PF in an alkaline matrix. The advantages of PF are obvious: wide source, low price, energy saving and environmental protection. However, the structure and properties of PF lead to degradation of the alkali matrix. Improving the durability of PF-reinforced geopolymers and slowing matrix degradation are key factors in their large-scale engineering applications. The main findings are as follows. The hygroscopicity of PF affects its mechanical properties. However, this property can be used to achieve curing efficiency in the matrix of composite materials to improve the strength of the matrix. There is a certain degree of alkaline degradation of PF in the geopolymer matrix, and the degree of degradation is closely related to the alkalinity of the geopolymer. Under the influence of calcium hydroxide, PF causes both degradation and mineralization of the cement matrix. Compared with the cement matrix, the geopolymer matrix significantly slows PF degradation. The most direct way to reduce PF degradation in the matrix is to further reduce the pH value and calcium hydroxide content of the matrix. Alkaline degradation of PF in the matrix has an adverse effect on the mechanical properties of composite materials, and the impact of fiber degradation can be mitigated by chemical modification. Nanomaterials can improve the microstructure of the composite matrix, accelerate the polymerization reaction of the matrix, increase the amount of matrix gel, improve the density of the matrix, improve the bond between the fiber and matrix, and thus reduce the rate of PF deterioration. In fact, the PF used in the matrix is classified as recycled and virgin. Straw fibers and coir fibers are waste. The full use of waste fibers is of environmental importance. In addition, replacing cement with geopolymer is an effective way to slow down the degradation of PF in the matrix. At the same time, a low-base activator replacement should be used in the geopolymer matrix. To date, there have been relatively few quantitative studies on the proportion of geopolymer mixture affecting fiber degradation, so this should be one of the research directions in the future^37^.

An analysis of the benefits and limitations associated with the addition of fibers to geopolymer matrices, as well as the desire to develop an environmentally friendly solution for the construction industry as an innovative construction and insulation material, led to the realization of a study of multi-layer geopolymer composites with natural fiber insulation mats. Thanks to the use of layered composites where the fiber layers are in the form of an agglomerate of considerable thickness, it is possible to reduce the degradation of natural fibers due to the contact of the matrix only with the surface layer of the applied mats. Degradation in such a case, if it occurs, takes place only for a small number of applied fibers. The use of natural fibers, which are a renewable material, brings environmental benefits, as these materials are an alternative to the commonly used plastic insulation or to mineral wool. Tests carried out prove that the insulation levels of such mats and felts made of natural fibers do not differ from the insulation levels for commonly used materials (the same range of thermal conductivity coefficient). In addition, the manufacture of multi-layer composites brings additional benefits in the form of improved insulating performance due to the presence of multiple resistances at the boundary between the individual phases/materials, which is also confirmed by the studies of other authors^38,39^.

Multi-layer geopolymer materials based on anthropogenic raw materials and fibers of natural origin can, if implemented, become an answer to the existing problems of the modern world related to the need to tighten requirements in construction as a result of reducing CO_2_ emissions. Multi-layer construction is characterized by a shell consisting of several high-strength outer layers and one or more low-density inner layers^40^. Geopolymers are a better alternative to popular Portland cement-based materials, as confirmed by numerous publications. The process of manufacturing geopolymers is much more economical and environmentally friendly due to two aspects. It is estimated that during the production of geopolymers there are up to 10 times lower emissions of CO_2_ and other greenhouse gases, and 4–8 times less electricity is consumed than in the case of traditional building materials using Portland cement. They are also materials with a much lower carbon footprint and fit into a closed-loop economy. These materials have similar properties to commercially available products, but when it comes to climate protection, they are much more environmentally friendly and energy efficient. The use of natural fibers is another strong emphasis on environmental friendliness, for the reason that fibers are a readily available and renewable material, and their properties are similar or better to/from existing insulation solutions in construction^41–43^.

The purpose of this publication was to conduct basic research on natural materials of plant origin and fly ash, the base raw material for the manufacture of multi-layer geopolymer composites. The finished prototype materials are also presented and their basic physical and thermal properties are given, which are key to optimizing the new solution. It should also be noted that this is the first article in the series and the basic part of the research will be presented here, and the topic, due to the fact that it is very extensive, will be continued in subsequent publications. The matrix material of the new composite was a geopolymer obtained by alkaline activation of aluminosilicates (synthesis), while plant fibers of natural origin were used as the material for the insulating layers. The prototype material was created through a pouring method, where two types of fibers were placed inside as mats. The new proposed construction solution is an undoubted innovation, being more energy-efficient and environmentally friendly than the conventional construction materials used so far. The multi-layer geopolymer composite is a material that can be manufactured in the prefabrication process. The innovativeness of the proposed solution lies not only in the use of fibers in geopolymers that are agglomerated in several layers, but also in the reinforcement of all layers through the use of resilient fiberglass bars to prevent possible decomposition. With this new solution we have a number of advantages such as:

- Use of low-carbon binders with less adverse environmental impact.
- Prevention of fiber degradation in the matrix matrix.
- Prevention of decomposition (delamination) of multi-layer composites.
- Significant reduction in thermal conductivity due to the creation of interfacial resistances.
- Elimination/replacement of commonly used insulating materials in favor of renewable materials.

## Materials and methods

**Figure 2:**
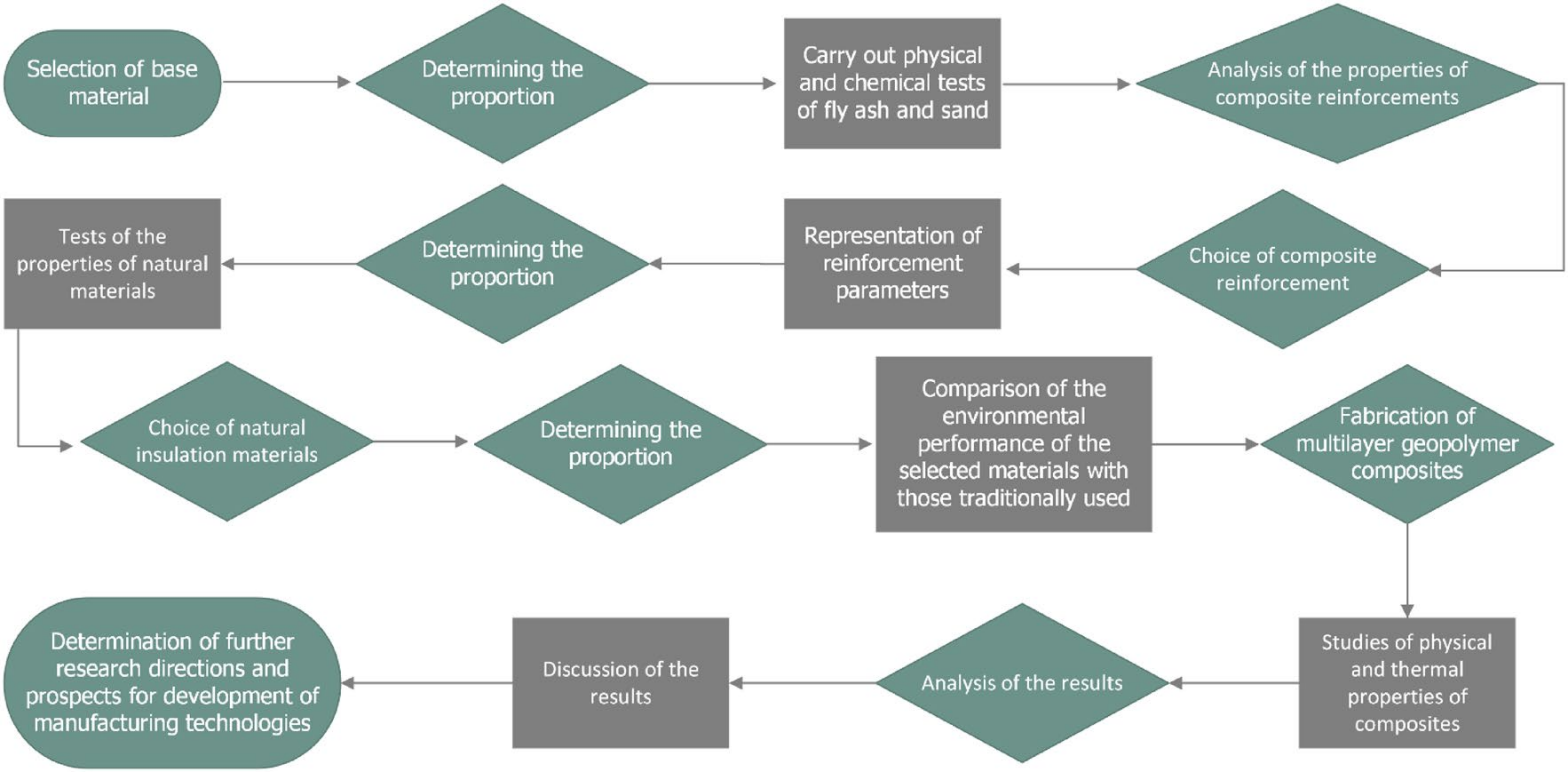
Experimental flowchart of the steps carried out in the methodological and research parts.

In order to understand the methodological procedure in this article, below in Fig. 2 is an experimental flow chart of the steps carried out in the methodological and research sections.

### Base materials

Multi-layer geopolymer composites were produced based on fly ash from the Skawina Heat and Power Plant (Skawina, Poland). Class F fly ash with certificate number 1488-CPR-0166/W was used. Based on the aforementioned document, the key parameters of this anthropogenic waste were also given, including the loss of roasting was classified into categories A and B, the fineness—category N (declared value of 20%), while the grain density of this material was 2210 kg/m^3^. This ash has a high content of SiO_2_ (silicon dioxide) and more than half the content of Al_2_O_3_ (diglinium trioxide). The second base material of the composite was sand from the Świętochłowice Sand Plant (Świętochłowice, Poland). Both of these materials were added in a 50/50 weight ratio. The polycondensation reaction of the geopolymer was initiated by adding a 10-mol solution of alkaline sodium silicate with sodium water glass. Sodium silicate R-145 with a molar modulus of 2.5 and a density of about 1.45 g/cm^3^ was used.

### Reinforcement

To make the finished structural element stable and strong, and to make the multi-layer composite monolithic and not delaminate during external interference, composite bars (4 and 8 mm in diameter) coated with glass fiber and infused with epoxy resin (TROKOTEX, Poland) were used. The characteristics of the physical and chemical properties of these bars are shown in Table 1^44^.

**Table 1:** Physicochemical properties of glass fiber saturated with epoxy resin.

Traits studied	Value
Tensile strength (MPa)	1250
Elasticity modulus (MPa)	55,000
Susceptibility to deformation	Linear-elastic
Linear expansion coefficient (%)	2.2
Thermal conductivity coefficient (W/m*K)	0.35
Density (kg/m^3^)—Specific gravity (N/m^3^)	1890
Corrosion resistance	Completely corrosion resistant
Electrical conductivity	Dielectric
Impact on electromagnetic wave interference	Transparent to waves
Eco-friendliness	Eco-friendly, easy to dispose of
Durability	min. 100 years

### Natural materials of plant origin

Before the two composite core materials were selected, several variants of natural fibers were tested for their key parameters. Tests were conducted for 9 types of natural fibers. Two instead of cellulose (PRO AGRO, VestaEco, Poland), hay and wood shavings (supplier Dach-Wkręt, Poland), coconut fiber from Sri Lanka (producer—PROMAT, Poland) and flax and hemp fiber and 3 types of hemp fiber (Double Raw Fiberworks, Poland) were used. In addition to natural fibers, mats, felts, and wools of natural origin were also tested. The coconut mat came from the upholstery wholesaler Akces (Strażów, Poland). Other felts and wools, on the other hand, were purchased from Double Raw Fiberworks (Cracow, Poland). Nine different types of fibers were selected for the study to compare the most important physical and thermal parameters, as well as the structure and texture of the components. Most of these fibers are widely available on the market, while hay and wood shavings were chosen because they have not been used in insulating building envelopes before. As for mats, felts, and wools, materials that contained the fibers discussed above were selected because the essence of the fibers' performance and properties are different, and finished products widely available based on these fibers behave differently. A market reconnaissance was conducted to select and compare multi-criteria fibers, mats, wools, and felts in terms of availability, thermal conductivity, environmental performance, and price.

### Eco-friendliness and energy efficiency of materials

Since the newly developed material is intended to be environmentally friendly and energy efficient, an analysis was also carried out from this angle. Table 2 shows data on embodied energy (EE) and embodied carbon (EC). These values are determined using the Life Cycle Analysis (LCA) method. Life Cycle Analysis (LCA) is a method used to assess the environmental impact of a product throughout its life cycle, including extraction and processing of raw materials, production, distribution, use, recycling, and final disposal. In conventional buildings, operational energy is closer to total energy and embodied energy is relatively low; low-energy buildings have a higher proportion of embodied energy to total energy; passive buildings have equal operational and embodied energy; and finally, self-sufficient or plus-energy buildings have no operational energy, and the total energy considered in the LCA is embedded energy (total energy is higher than in passive houses)^45–47^.

**Table 2:** Embodied energy and embodied carbon for building and insulation materials.

Material	EE (Mj/kg)	EC (kgCO_2_/kg)
Fly ash	0.1	0.01
Sand	0.1	0.005
Cement	4.6	0.83
Concrete	0.95	0.13
Geopolymer	0.33	–
Cellulose	0.94–3.3	–
Flax (insulation)	39.5	1.70
Mineral wool	16.6	1.20
Rock wool	16.8	1.05
Paper wool (wood shavings)	20.17	0.63
Wood wool	10.8	–
Recycled wool	20.9	–
Wool (overall)	3	0.15
Foamed polystyrene	88.6	2.50
Thermoformable foamed PS	109.2	3.40
Polyurethane	72.1	3.00
Coconut mat	42	–
Flax mat	50	–
Hemp mat	40	–

### Methods

#### Density measurements

The density of the finished composite plates was forfeited using the geometric method, based on the mass and volume of the samples. The density of bodies with regular shapes is determined according to the formula:

$$ d = \frac{{\text{m}}}{{\text{V}}} \left( {\frac{{{\text{kg}}}}{{{\text{m}}^{3} }}} \right) $$

where m—regular-shaped body weight (kg), V—regular-shaped body volume (m^3^).

#### Thermal conductivity measurements

For a heat-conducting cuboidal body under steady-state conditions, the amount of heat transferred is substance-dependent, proportional to the cross-section of the body, the temperature difference and the time of heat flow. This quantity is determined according to the formula:

$$ \lambda = \frac{{\text{Q}}}{{\text{t}}}*\frac{{\text{d}}}{{{\text{S}}\Delta {\text{T}}}} \left( {\frac{{\text{W}}}{{{\text{m}}*{\text{K}}}}} \right) $$

where Q—amount of heat flowing through the body (J), t—flow time (s), d—partition thickness (m), S—cross-sectional area of the body (m^2^), $\Delta \text{T}$—temperature difference in the direction of heat conduction (K).

### Ready-made composites

**Figure 3:**
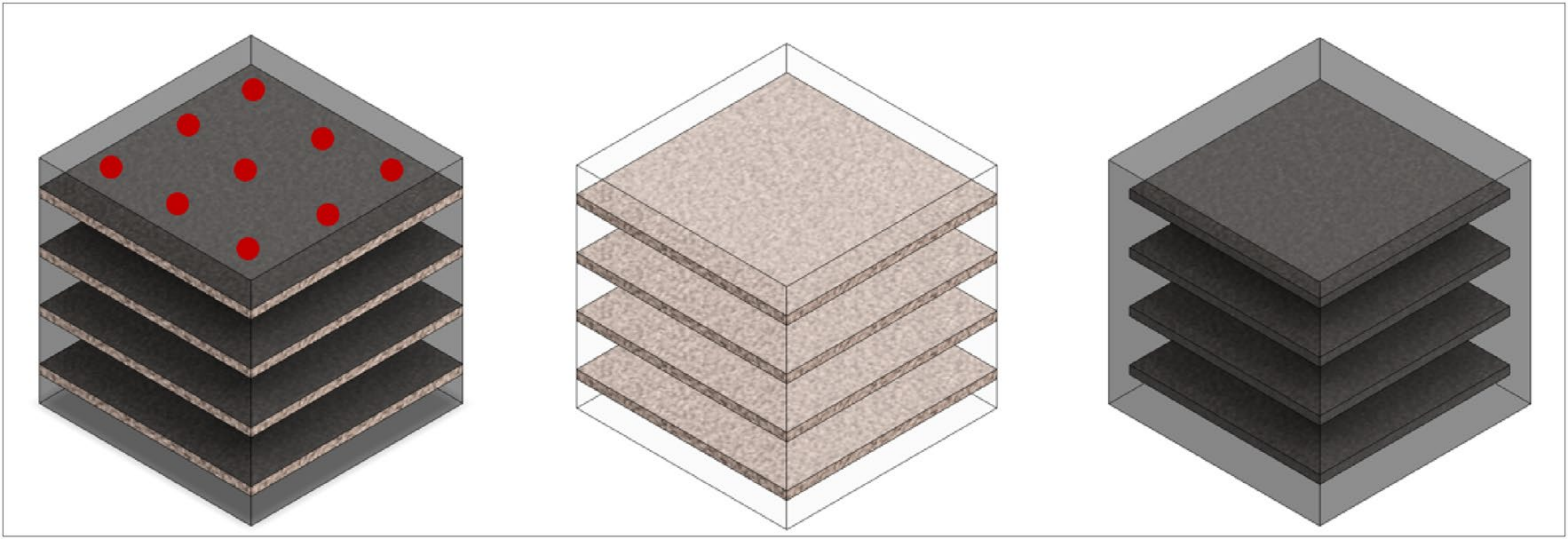
Model of multi-layer geopolymer composite: (**a**) composite, (**b**) layers of natural insulation, c) geopolymer layer.

After researching the properties of natural materials, two composite core materials were selected: coconut mat and flax-hemp felt 1. Both of these materials were chosen because of their similar density and thermal conductivity coefficient, as well as the high availability of these materials on the market. Coconut mat is often used in mattresses, but the use of this material as insulation in building envelopes has not been reported so far, hence the idea to use it in a completely different industry. The other material has similar properties to coconut mats hence the idea to compare the performance of these materials in multi-layer partitions. As mentioned above, the multi-layer geopolymer composites were made from fly ash and sand. Reinforcing bars were also added to increase the strength of the finished precast prototype. To each geopolymer composite were added 9 bars with a length of 15 cm (diameter 4 and 8 mm) and 4 layers of natural materials each. Two types of bars were used to compare the effect on the insulation performance of the finished composites. Due to the different thickness and weight of the mat and felt, a different weight amount of the component was introduced into the composite. The density of the two materials is similar, but physically and visually they differ significantly. Table 3 shows the characteristics of the finished multi-layermulti-layer composites (with dimensions of 15 × 15x15 cm). The table shows the weight and weight share of each component. In the remainder of the article, the properties of the composites will be compared in terms of the additive introduced and the total weight shares of the components (natural insulation and bars). The table below shows that compared to the reference composite, the amount of introduced additives is very low. Figure 3, on the other hand, shows a model of a multi-layer geopolymer composite. The bars are marked in red.

**Table 3:** Characteristics of finished multi-layer composites.

Material	Cube weight (g)	Fibers weight (g)	Fibers share (wt%)	Bars weight (g)	Bars share (wt%)
reference	5746.0	0	0	0	0
4 flax-hemp felts (4 mm bars)	5514.0	55.4	1	44.6	1
4 coconut mats (4 mm bars)	4831.2	121.6	3	44.6	1
4 flax-hemp felts (8 mm bars)	5216.8	59.7	1	121.5	2
4 coconut mats (8 mm bars)	4749.6	119.8	3	121.5	3

## Results

### Testing of base materials

Oxide X-Ray Fluorescence chemical composition analysis was performed for the base materials, namely fly ash and sand. The oxide XRF analysis was performed on a SCHIMADZU EDX-7200 (SHIMADZU Europa GmbH, Duisburg, Germany). The test was carried out in an air atmosphere with holders designed for bulk materials and Mylar film, and the results are shown in Tables 4 and 5.

**Table 4:** Oxide analysis for fly ash.

Precursor	Oxide composition (wt%)					
	SiO_2_	Al_2_O_3_	K_2_O	FeO	Na_2_O	MgO
Fly ash	57.84	32.46	4.75	1.78	1.61	1.57

**Table 5:** Oxide analysis for sand.

Precursor	Oxide composition (wt%)					
	SiO_2_	Al_2_O_3_	FeO	CaO	K_2_O	MgO
Sand	92.09	3.32	3.26	0.50	0.41	0.41

For the production of geopolymers, raw materials are used that are characterized by a high content of silicon and aluminum oxides. The above analysis of chemical composition shows that both fly ash and sand have high contents of these oxides. Fly ash typically contains 50–60 wt% SiO_2_ and about half that of Al_2_O_3_. Sand usually has a high SiO_2_ content (about 85–95 wt%) and secondarily usually has about 4 wt% Al_2_O_3_. Geopolymers are, according to the commonly accepted definition, inorganic, amorphous, synthetic aluminosilicate polymers formed from the synthesis of silicon (Si) and aluminum (Al) and geologically sourced minerals. Both of these materials thus fit into the idea of geopolymer materials.

**Figure 4:**
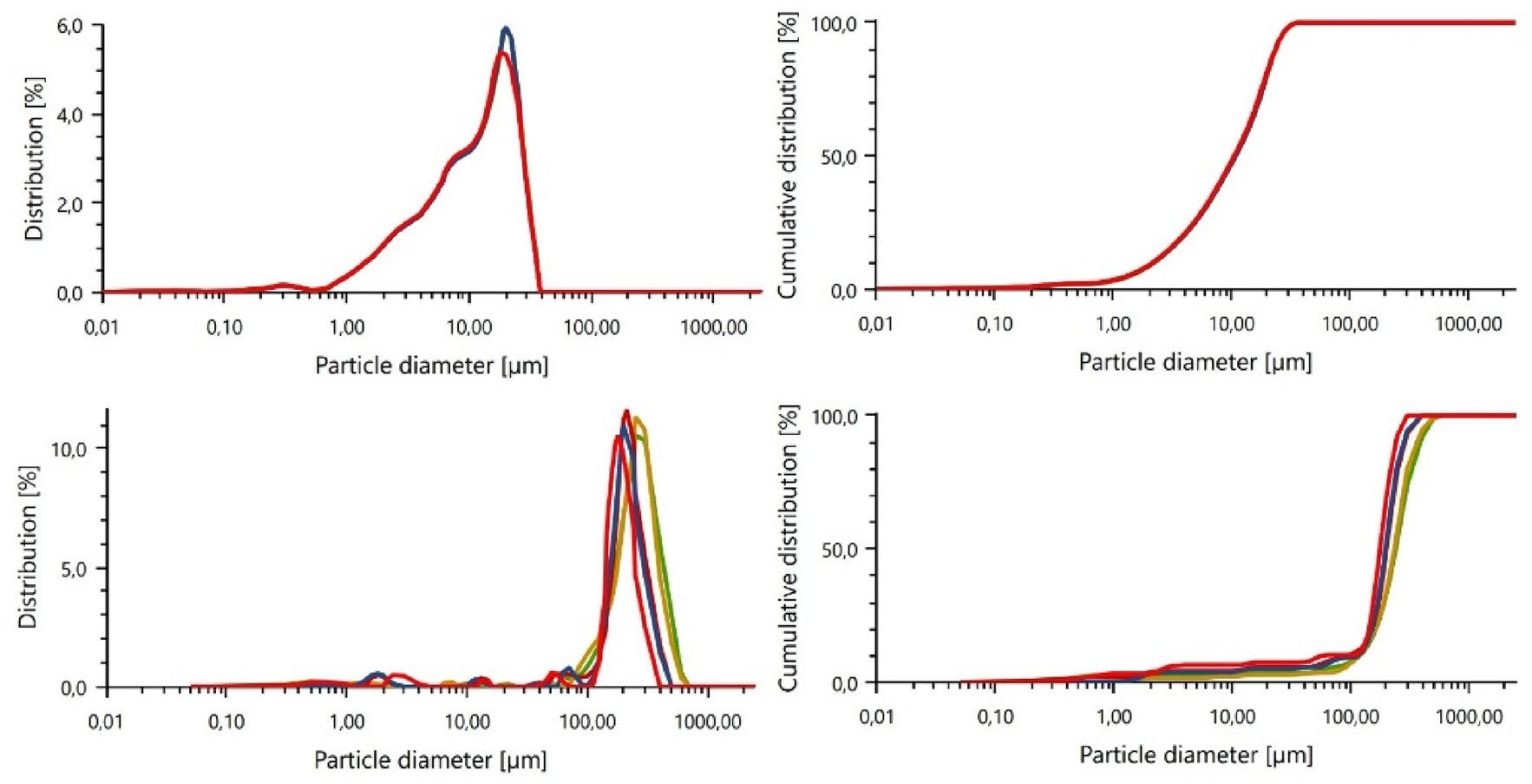
Particle size analysis of base materials: (**a**) fly ash particle size distribution, (**b**) fly ash cumulative curve, (**c**) sand particle size distribution, (**d**) sand cumulative curve.

Using an Anton-Paar PSA 1190LD particle size analyzer (AntonPaar GmbH, Graz, Austria), particle size analysis of the two base materials was also performed using Kalliope Professional software (version 2.22.1). Particle size analysis for fly ash was carried out wet (water was the dispersing agent), while for sand this analysis was performed dry, due to the very large particle size. Wet analysis should be used for low particle-size materials, while dry analysis is recommended for large material particles. Particle size measurements of each material were taken 5 times and the particle size distribution and cumulative curve are presented. The results of the measurements are shown in Fig. 4.

Fly ash is typically a very fine material, as confirmed by particle size analysis. The average particle size for this material is about 13 µm. Sand tends to have a large particle size, which was also confirmed by this analysis. The sand particles had an average particle size of 220 µm. Both of these materials react very well with each other when an alkaline activator is added and show the ability to form a monolithic structure, similar to Portland cement-based concretes and mortars.

### Testing of natural materials of plant origin

**Figure 5:**
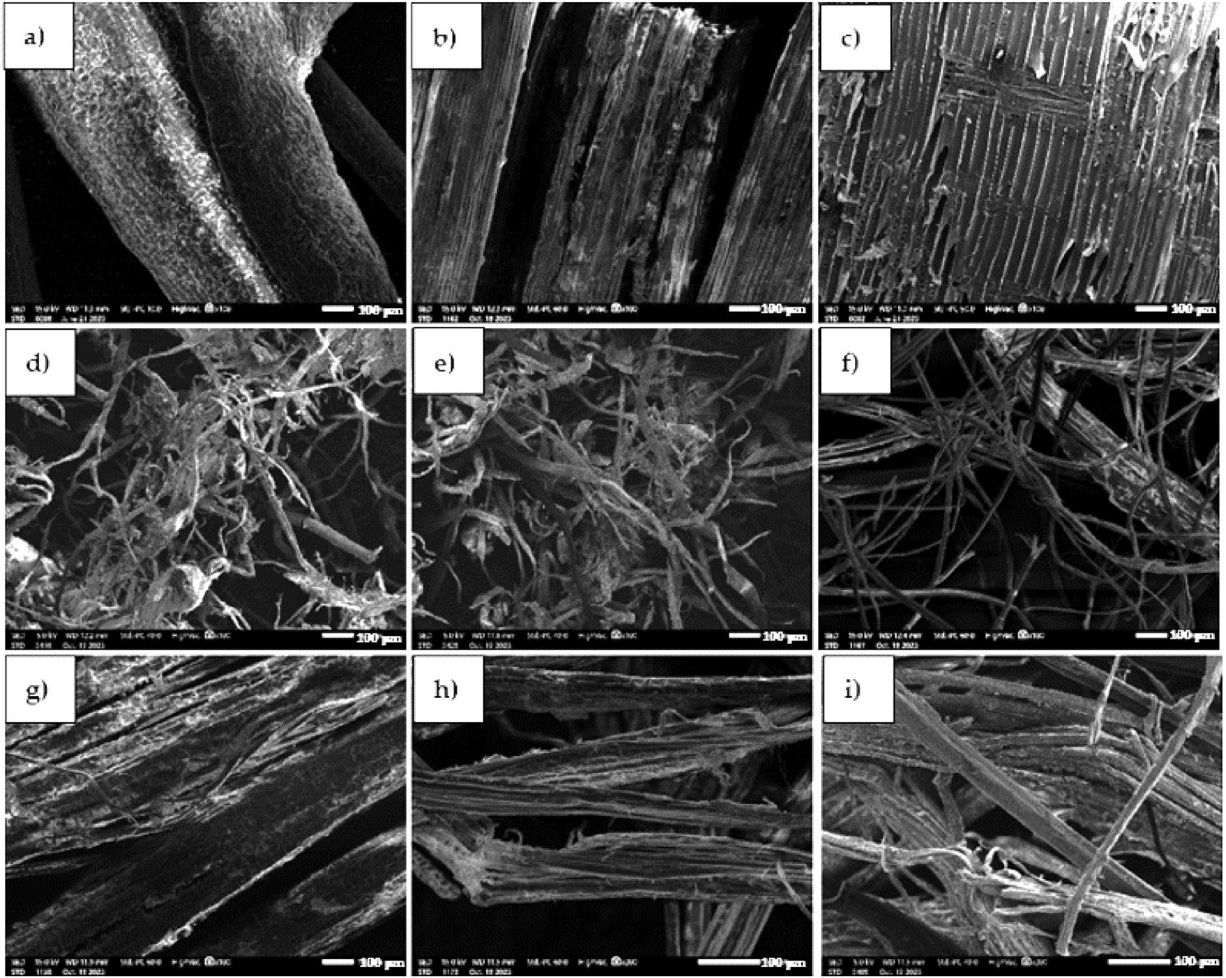
Microscopic images of fibers: (**a**) coconut fiber, (**b**) hay, (**c**) wood shavings, (**d**) PRO AGRO cellulose, (**e**) VestaEco CELL cellulose, (**f**) flax-hemp fiber, (**g**) hemp fiber 1, (**h**) hemp fiber 2, (**i**) hemp fiber 3.

**Figure 6:**
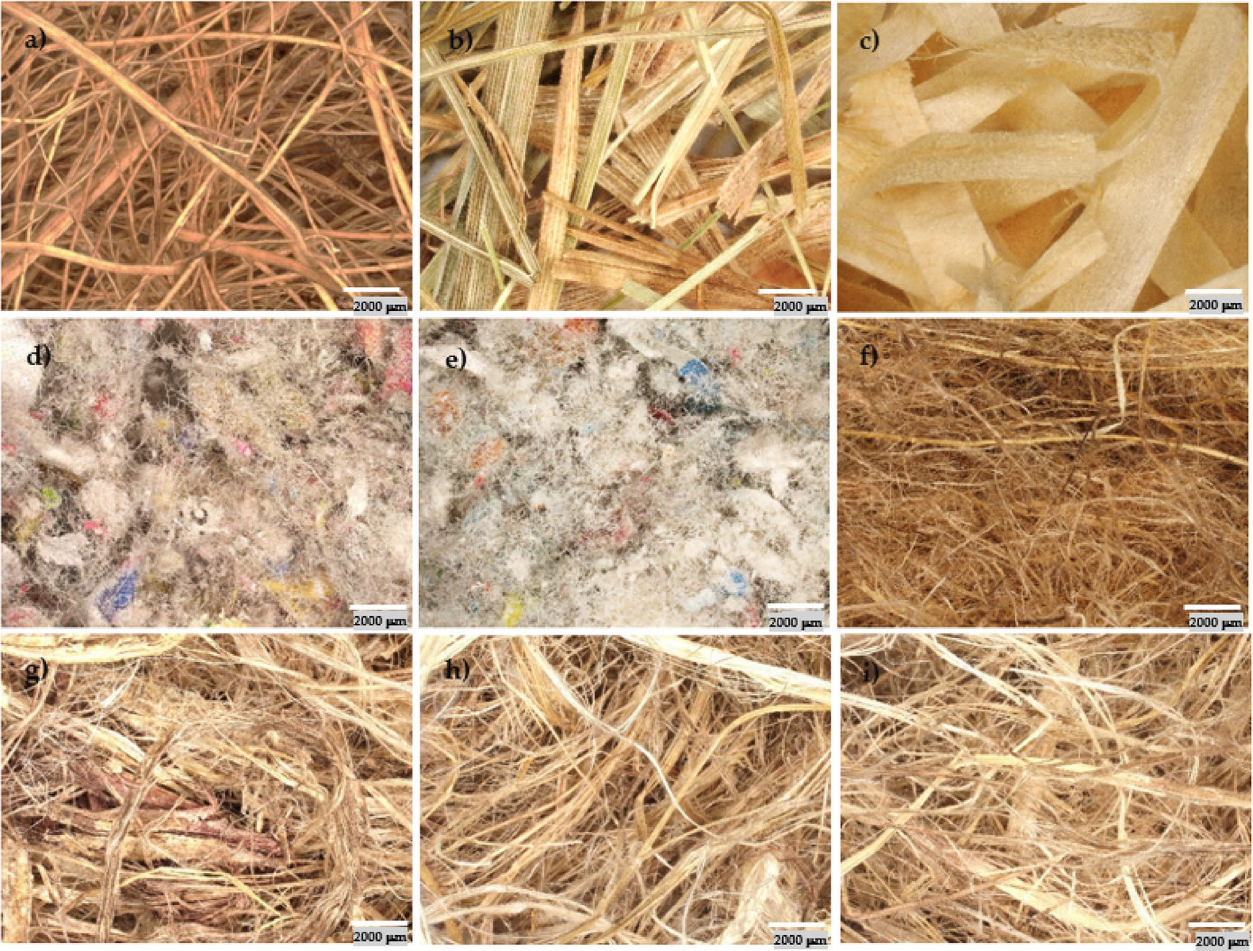
Macroscopic images of fibers: (**a**) coconut fiber, (**b**) hay, (**c**) wood shavings, (**d**) PRO AGRO cellulose, (**e**) VestaEco CELL cellulose, (**f**) flax-hemp fiber, (**g**) hemp fiber 1, (**h**) hemp fiber 2, (**i**) hemp fiber 3.

For the natural fibers, density measurements and thermal conductivity tests were performed on an HFM Lambda 446 plate apparatus (NETZSCH Pumpen & Systeme GmbH, Waldkraiburg, Germany) and images were taken using scanning electron microscopy SEM on a JEOL IT 2000 microscope (JEOL, Akishima, Tokyo, Japan) and macroscopic images on a Keyence VHX-7000 digital optical microscope (KEYENCE INTERNATIONAL, Mechelen, Belgium). In order to carry out the observation of the microstructure, the fibers were attached to special carbon disks and placed on metal tables and then in a holder. A special EM-Tec C33 carbon glue was also used to better attach the fibers and lead to better conduction of the material. Before testing, the surface of the fibers was coated with a conductive gold layer using a DII-29030SCTR Smart Coater vacuum sputtering machine (JEOL Ltd., MA, USA). The authors only analyzed the morphology of the samples; the chemical composition of the fibers was not studied. Table 6 shows the results of density and thermal conductivity (average value from 3 measurements), while Figs. 5 and 6 show microscopic photos and macroscopic photos of the fibers. The microscopic photos were taken at 100× magnification, while the macroscopic photos were taken at 20× magnification.

**Table 6:** Parameters of natural plant-based fibers.

Fibres	Density	Thermal conductivity
	(kg/m^3^)	(W/m × K)
Cellulose PRO AGRO	52.06	0.03966
Cellulose VestaEco CELL	54.11	0.03976
Coconut fibers	47.15	0.04667
Flax-hemp	51.29	0.03926
Hay	46.35	0.04239
Hemp 1	39.20	0.05211
Hemp 2	35.75	0.04484
Hemp 3	34.23	0.04528
Wood shavings	34.06	0.04424

Tests on the density and thermal conductivity of natural materials showed that wood shavings had the lowest density because they were the lightest. In contrast, PRO AGRO cellulose had the highest density. As for thermal conductivity, the lowest result was recorded for flax-hemp fiber, while the highest was for hemp fiber 1. All natural fibers had a density of 34–52 kg/m^3^ and a thermal conductivity coefficient of 0.039–0.052 W/m*K. Studies of the morphology of the structure and texture of the natural fibers on scanning and optical microscopy showed that the fibers are completely different from each other. Coconut fiber is characterized by a spongy structure, which provides very good moisture absorption, air access, and a durable structure. Hay and wood shavings have a uniform band structure. Cellulose fibers have a fibrous structure that differs significantly in shape and arrangement. Flax fiber has an irregular highly porous structure. Hemp fibers, on the other hand, have an open-pore cell structure. They can absorb moisture and release it again after drying. Macroscopic images are shown to illustrate the appearance of the fibers. The SEM images show the microstructure of the fibers, while the digital optical microscope images show the actual appearance of the fibers at low magnification.

In addition to natural fibers, mats, felts and wools made from the fibers shown above were also tested. Results for 10 different materials are presented, and each material, due to the fact that they are used in layered partitions, was tested as 1, 2 and 3 layers of material. The density and thermal conductivity of these materials are shown in Table 7 (again, average values from 3 measurements are shown).

**Table 7:** Parameters of mats, felts and wools of plant origin.

Material	Density	Thermal conductivity
	(kg/m^3^)	(W/m × K)
Coconut mat_1 layer	123.86	0.03825
Coconut mat_2 layers	121.6	0.04048
Coconut mat_3 layers	126.9	0.04124
Jute felt_1 layer	84.91	0.03368
Jute felt_2 layers	84.80	0.03625
Jute felt_3 layers	82.11	0.03636
Flax felt_1 layer	113.50	0.03364
Flax felt_2 layers	120.80	0.03635
Flax felt_3 layers	123.27	0.03753
Flax-hemp felt 1_1 layer	121.98	0.03928
Flax-hemp felt 1_2 layers	118.65	0.03985
Flax-hemp felt 1_3 layers	121.25	0.04295
Hemp felt_1 layer	116.69	0.03547
Hemp felt_2 layers	121.59	0.03823
Hemp felt_3 layers	121.42	0.03938
Flax-jute felt_1 layer	106.54	0.03677
Flax-jute felt_2 layers	107.65	0.03814
Flax-jute felt_3 layers	104.02	0.03890
Flax-hemp felt 2_1 layer	99.49	0.03568
Flax-hemp felt 2_2 layers	103.44	0.03636
Flax-hemp felt 2_3 layers	115.82	0.04037
Linen wool (pressure)	78.85	0.03101
Linen wool (no pressure)	14.06	0.04303
Hemp wool (pressure)	53.71	0.03315
Hemp wool (no pressure)	13.85	0.04799
Flax-jute wool (pressure)	38.75	0.03529
Flax-jute wool (no pressure)	14.40	0.04725

Tests on the density and thermal conductivity of mats, felts and wools showed that the lowest density was achieved by hemp wool without pressure, because there are very large air voids in the material. On the other hand, the highest density was achieved by the coconut mat, which, of all the components, was the heaviest. As for thermal conductivity, linen wool without pressing had the lowest result, while hemp wool without pressing had the highest. All mats, felts and wools had a density of 14–127 kg/m^3^ and a thermal conductivity coefficient of 0.031–0.047 W/m*K. Two materials were selected for further study: flax-hemp felt 1 and coconut mat. Both of these materials have similar density and thermal conductivity.

### Density of ready made composites

The density of the panels was calculated three times and an average value was calculated from the three measurements, which is given in Table 8. The volume of the panels was constant (15 × 15 × 5cm), but the weight varied slightly from sample to sample. The dimensions of the samples were measured using a laboratory caliper with an accuracy of 0.01 mm, and the weight of the samples was determined using a RADWAG PS 200/2000.R2 precision laboratory analytical balance with an accuracy of 0.01 g.

**Table 8:** Density of multi-layer panels.

Material	Mean density (kg/m^3^)
reference	1777.33
4 flax-hemp felts (4 mm bars)	1694.51
4 coconut mats (4 mm bars)	1448.23
4 flax-hemp felts (8 mm bars)	1546.14
4 coconut mats (8 mm bars)	1382.71

Each component caused a decrease in density, due to the fact that the introduced additives reduced the proportion of base materials. The largest decrease in density was observed for a panel with 4 coconut mats and 8 mm bars (22% decrease)—6 wt% components (natural insulation and bars). For a similar variant only with a smaller diameter of bars, a density reduction of 19% was obtained—4 wt% of components. The panel with 4 flax-hemp felts and 4 mm bars obtained a density decrease of 5%—2 wt% of the components, while the same sample only with 8 mm bars—a decrease of 13% 3 wt% of the components. In each multi-layer panel, a greater decrease in density was observed with the addition of 8 mm bars, because the larger bar area resulted in a reduction in the proportion of geopolymer mortar. The coconut mats were much higher than the flax- hemp felt, and therefore caused a greater decrease in density as the proportion of geopolymer again decreased.

### Thermal conductivity of ready made composites

The thermal conductivity coefficient measurements were carried out on a Lambda HFM 446 plate apparatus (Netzsch). The device operates according to standards such as ISO 8301, EN 12664, ASTM C1784, ASTM C518, and others. Temperature regulation and control are verified by a Peltier system. The thermal properties of the finished panels were determined using the described device based on the hot and cold panel method. Thermal conductivity was tested in three temperature ranges: 0–20 °C, 20–40 °C, and 30–50 °C to better illustrate the performance characteristics. In real conditions, the materials can operate at temperatures above 30 °C, hence the 3 different temperature ranges. Table 9 shows the average conductivity based on the three tests.

**Table 9:** Thermal conductivity coefficient of multi-layer panels.

Material	λ at 0–20 °C (W/m × K)	λ at 20–40 °C (W/m × K)	λ at 30–50 °C (W/m × K)
Reference	1.36511	1.36368	1.38825
4 flax-hemp felts (4 mm bars)	1.14216	1.16104	1.17736
4 coconut mats (4 mm bars)	0.87829	0.88672	0.89940
4 flax-hemp felts (8 mm bars)	0.86741	0.88275	0.90070
4 coconut mats (8 mm bars)	0.80541	0.81783	0.82380

Each component, as in the previous study, caused a decrease in the thermal conductivity coefficient, and thus improved the insulation performance. The largest decrease in thermal conductivity was observed for the panel with 4 coconut mats and 8 mm bars (41% decrease)—6 wt% components (natural insulation and bars). Very similar results were obtained by adding 4 coconut mats (4 mm bars) to the composite—4 wt% of the components, and 4 flax-hemp felts (8 mm bars)—3 wt% of the components. A 36% decrease in lambda coefficient was achieved for these panels. Components of similar density and conductivity were introduced into the geopolymer matrix, but the results show that lower conductivity was obtained for the samples with 8 mm bars. The composite bars had a thermal conductivity coefficient of 0.35 W/m × K, while solid geopolymer typically has conductivities close to 1–1.4 W/m × K. Introducing a component with low thermal conductivity and a larger surface area (8 mm) into the geopolymer matrix than the 4 mm diameter bars resulted in better results, as the proportion of geopolymer decreased. And again as in the case of density, the larger surface area of the coconut mat resulted in a reduction in the proportion of geopolymer, thus improving the insulating capacity of the structure. As for the difference between the temperature ranges, it is of the order of 0.01–0.02 W/m × K.

## Discussion

This study analyzed the physicochemical properties of the materials that serve as precursors for the geopolymer matrix, as well as the physical and thermal properties of the natural materials that serve as insulating layers. Qualitative image analysis was also carried out for the natural materials using a digital optical microscope and a scanning electron microscope. After conducting basic research, prototypes of prefabricated multi-layer composites were designed and their physical and insulating properties were tested.

The prototypes used composite bars, specifically fiberglass bars infused with epoxy resin. These offer a superior alternative to steel bars and prevent delamination of multi-layer composites. This material was chosen for its several key advantages. It is an attractive solution for construction in combination with multi-layer applied natural fiber mats and felts. The thermal expansion of composite reinforcement is comparable to concrete and excludes the formation of cracks in concrete under temperature changes, eliminating the cost of repairing cracks during the operation of the building. Glass fiber reinforcement is completely resistant to corrosion, does not change its properties even in highly corrosive and aggressive acid and alkaline environments, so it does not require tedious and expensive maintenance. These bars are also characterized by high frost resistance and 2–3 times longer material life^44^.

Data on embodied energy of materials and carbon dioxide were also analyzed. Fly ash, sand, and geopolymers have significantly better environmental performance than conventional materials such as cement or concrete. Geopolymer has almost 3 times lower embodied energy than concrete. Fly ash has 46 times lower embodied energy and 83 times lower embodied carbon dioxide than cement. When it comes to insulation, polystyrene or urethane foams also fare worse in this comparison than materials or natural fibers. Each of the natural materials analyzed has better environmental performance than conventionally used foams (up to 5–10 times)^45–47^.

Analysis of the properties of natural materials showed that the lowest density was achieved by wood shavings because they were the lightest. In contrast, PRO AGRO cellulose had the highest density. Of all the natural fibers, flax and hemp fiber had the lowest thermal conductivity, and hemp fiber 1 had the highest. All fibers had thermal conductivities in the range of 0.039–0.052 W/m*K. Analysis of the results for mats, felts and wools showed that the lowest density was achieved by hemp wool without pressure because there are very large air voids in the material. In contrast, the highest density was achieved by the coconut mat, which was the heaviest of all the components. Linen wool had the lowest thermal conductivity coefficient among the tested materials, and hemp wool had the highest. All results oscillated between 0.031 and 0.047 W/m*K. Tests were conducted on several layers of the material to see how this material would behave in layered composites. The thickness of the material affects its insulating performance, so the worst results were obtained for 3 layers of material, but these are not results that would preclude their use as an alternative to existing insulating materials. Since all of these materials are biodegradable and renewable, it can be concluded that they are a better choice as an addition to sandwich partitions compared to conventional insulation. Other researchers have obtained similar results for the density and conductivity of natural materials^48–50^.

Studies of the physical properties of the composites showed that each component caused a decrease in density, due to the fact that the introduced additives reduced the proportion of base materials. The largest decrease in density was observed for a panel with 4 coconut mats and 8 mm bars (22% decrease). In this composite, 6 wt% of the components (natural insulation and reinforcement) were introduced and thus the best result was obtained. For a similar variant only with a smaller diameter of bars, a 19% reduction in density was achieved—4 wt% of components were introduced. The panel with 4 flax-hemp felts and 4 mm bars obtained a decrease in density of 5% (2 wt% of components), while the same sample only with 8 mm bars—a decrease of 13% (3 wt% of components). In each multi-layer panel, a greater decrease in density was observed with the addition of 8 mm diameter bars, because the larger bar area resulted in a reduction in the proportion of geopolymer mortar. The coconut mats, being thicker than the flax-hemp felt, displaced more geopolymer, resulting in a greater decrease in density. A decrease in density after the introduction of lower density components is a natural physical phenomenon and has been confirmed more than once^51,52^.

Analysis of the thermal parameters of the newly formed composites showed that the largest decrease in thermal conductivity was observed for the panel with 4 coconut mats and 8 mm bars (41% decrease)—6 wt% components were introduced. Very similar results were obtained by adding 4 coconut mats (4 mm bars) to the composite—4 wt% of components, and 4 flax-hemp felts (8 mm bars)—3 wt% of components. A 36% decrease in lambda coefficient was achieved for these panels. Components with similar densities and conductivities were introduced into the geopolymer matrix, but the results show that a lower conductivity was obtained for the samples with 8 mm bars, because the larger bar area reduced the weight share of geopolymer. And again as in the case of density, the larger surface area of the coconut mat resulted in a reduction in the proportion of geopolymer, thus improving the insulating capacity of the structure. The decrease in thermal conductivity after the introduction of lower density components is a natural physical phenomenon and has been confirmed by many authors^53,54^.

The results of the research presented in this paper, confirm the results of other authors of scientific papers, that geopolymers are an attractive material for the creation of multi-layer structural-insulation composites^55,56^. The presented research results and the presented concept are an innovative contribution to the development of sustainable construction. So far, few authors have presented similar solutions. This topic will be continued and intensive work is currently underway to develop this technology for the manufacture of geopolymer composites for construction applications.

In the following work, studies will be presented to illustrate the impact of the distribution and orientation of natural materials in the geopolymer matrix. A key issue will be the optimization of the process and parameters of the produced composite. The author’s main goal is to create a material with the longest possible durability, which can last for decades without the need for renewal and repair. The work will be aimed at comparing how the distribution and form of the fibers affect the insulation and strength parameters. In addition, future work should focus on two key issues:

1. Create a solution that is easily applicable. The applicability of the conducted research should be confirmed in several ways. The created solution must be reproducible, and the planned products in the form of building partitions must be possible to produce in prefabrication. The method of manufacturing this type of composite may cause technological difficulties due to the large number of layers.
2. Study the effect of the number of layers formed on the thermal conductivity coefficient of the entire partition. It should be experimentally confirmed and proven that the use of the same volume or weight amount of insulating materials in the form of agglomerated natural fibers gives better insulating effects when these materials are present in several layers instead of one.

As a consequence of further planned research, an optimal insulating material will be created that will not only replace the building’s thermal insulation, but will also serve as a material for the construction of houses and structures that will no longer need to be insulated at a later stage. The target material will be tested for: thermal conductivity and heat penetration coefficient, strength, flammability, frost resistance, sound insulation and application use.

## Conclusions

Based on the research results presented in the article, several conclusions can be drawn to summarize the research work:

(I) Analysis of data on embodied energy of materials and carbon dioxide shows that fly ash, sand, and geopolymers have significantly better environmental performance than conventional materials such as cement or concrete. When it comes to insulation, polystyrene foam, or urethane, also performs worse in this comparison than materials or natural fibers.
(II) Wood shavings had the lowest density because they were the lightest. In contrast, PRO AGRO cellulose had the highest density. Analysis of the results for mats, felts and wools showed that the lowest density was achieved by hemp wool without pressure, because there were very large air voids in the material. In contrast, the highest density was achieved by the coconut mat, which was the heaviest of all the components.
(III) The lowest thermal conductivity among natural fibers was flax-hemp fiber, the highest was hemp fiber 1. Analysis of the results for mats, felts and wools shows that flax wool had the lowest conductivity, and hemp wool had the highest. All results oscillated between 0.031 and 0.052 W/m*K. Given that all of these materials are biodegradable and renewable, it can be concluded that they are a better choice as an addition to sandwich partitions compared to conventional insulation.
(IV) The largest decrease in density was observed for the panel with 4 coconut mats and 8 mm bars (22% decrease). In this composite, the largest number of components was introduced 6 wt%. The worst results were obtained for a panel with 4 flax-hemp felts and 4 mm bars (density decrease of 5%)—2 wt% components. Each component caused a decrease in density, due to the fact that the introduced additives reduced the proportion of base materials.
(V) The largest decrease in thermal conductivity was observed for a panel with 4 coconut mats and 8 mm bars (41% decrease)—6 wt% of components. The worst results were obtained for the panel with 4 flax-hemp felts and 4 mm bars (16% decrease in lambda conductivity)—2 wt% components. Each component again caused a decrease in the measured parameter.

Based on the above test results, it can be concluded that density and thermal conductivity measurements are closely related, which has been confirmed more than once by other scientists and specialized literature. The study showed that the worst test results were obtained for a panel with 4 flax-hemp felts and 4 mm bars. Geopolymers and natural fibers have better insulating properties and environmental performance than traditional building materials. Multi-layer geopolymer composites based on fly ash with the addition of natural fibers of plant origin are a novelty in the world of materials engineering, so the topic will be continued in the author’s future works. This topic has not yet been explored, and the idea presented in the article has a very high potential for commercialization.
